# The relationship between oxidized lipoprotein(a) and carotid atherosclerosis in asymptomatic subjects: A comparison with native lipoprotein(a)

**DOI:** 10.1186/1476-511X-10-174

**Published:** 2011-10-04

**Authors:** Kazuhiko Kotani, Shingo Yamada, Toshiyuki Yamada, Nobuyuki Taniguchi, Ikunosuke Sakurabayashi

**Affiliations:** 1 Department of Clinical Laboratory Medicine, Jichi Medical University, Tochigi, Japan; 2 Central Research Institute, Shino-Test Corporation, Kanagawa, Japan; 3 Saitama Memorial Hospital, Saitama, Japan

## Abstract

**Background:**

Oxidized lipoprotein(a) (oxLp(a)) can be a more potent marker of atherogenesis than native Lp(a), although Lp(a) is considered to be a risk factor for atherosclerotic diseases. Limited clinical data are available regarding the significance of oxLp(a) in atherosclerotic manifestations. This study aimed to investigate the association between the serum oxLp(a) and carotid artery intima-media thickness (CIMT), in comparison to the serum Lp(a) levels, among asymptomatic subjects.

**Methods:**

The atheroscrerosis-related variables including Lp(a) and oxLp(a) were measured in 136 cardiovascular disease-free subjects (61 males and 75 females, mean age of 64 years). The serum oxLp(a) level was quantified using a sandwich ELISA system. The CIMT level was ultrasonographically measured on bilateral carotid arteries.

**Results:**

The median level of Lp(a) was 120 μmol/L, oxLp(a) was 0.06 nmol/L, and CIMT was 0.7 mm, respectively. A simple correlation test showed that the CIMT was significantly and positively correlated with age, systolic blood pressure and oxLp(a) (r = 0.208, P < 0.05). A multiple linear regression analysis revealed that oxLp(a) continued to show a significant and positive correlation with the CIMT (β = 0.202, P = 0.01). Although the similar analyses were conducted for Lp(a), it showed only a weak correlation with the CIMT (r = 0.011, β = 0.041, both P < 0.05).

**Conclusions:**

These results suggest that oxLp(a) may be more closely associated with accelerated carotid atherosclerosis, in comparison to Lp(a), in this population. This finding can be important for obtaining a better understanding of the different atherogenic roles played by oxLp(a) in comparison to Lp(a).

## Introduction

The oxidation of lipids and lipoproteins is involved in the pathogenesis of atherosclerosis [1]. Lipoprotein(a) (Lp(a)) contains low-density lipoprotein (LDL)-like moieties, in which the apoB-100 component is covalently linked to the unique glycoprotein, apolipoprotein(a) (apo(a)), and a high circulating level of Lp(a) is considered to be a risk factor for atherosclerotic diseases [2,3]. While the physiological role of Lp(a) in atherosclerotic diseases remains incompletely understood, the oxidization of Lp(a) may be one of the crucial clues that atherogenesis has occurred. Of note, the existence of oxidized phospholipids on Lp(a) in the circulation has been reported to be strongly associated with coronary artery disease [4]. The earlier studies used an assay system that determines the content of oxidized phospholipids per particle of apoB-100 and primarily per Lp(a), and the results suggested that Lp(a) may participate in the transfer of oxidized phospholipids [4]. We have also identified oxidized Lp(a) (oxLp(a)) in human arteries and blood using an assay system which employs a unique antibody (161E2) to a specific epitope peptide of nine residues (Arg-Asn-Pro-Asp-Ala-Val-Ala-Ala-Pro) from the kringle-IV type 2 of apo(a) (this specific site is hidden on native Lp(a) particles, but appears on Lp(a) in the oxidative milieu) [5]. We have thus already accumulated some data using this oxLp(a) measurement [5-8].

Although earlier experimental studies have revealed that oxLp(a) can have more atherogenic properties than native Lp(a) [5,9-14], there have so far been only limited clinical studies using the serum oxLp(a) to investigate the association between oxLp(a) and atherosclerotic manifestations. More recently, two clinical studies have been published about this association [6,8]. One study reported that there was a significant and positive correlation between the serum oxLp(a) and pulse wave velocity index (a measure of systemic arterial stiffness) in hypertensive patients, and this correlation was relatively greater than that between the serum Lp(a) and pulse wave velocity index [6]. In addition, that study reported that the oxLp(a) deposition was histochemically detected, while the native Lp(a) deposition was not, in the coronary calcified areas in patients with myocardial infarction [6]. Another study reported that oxLp(a) was histochemically detected in endothelial cells, similar to native Lp(a), in human carotid and cerebral arteries, and the deposition of oxLp(a), but not native Lp(a), was abundant in the synthetic phase vascular smooth muscle cells in the same arteries [8]. These findings indicate that oxLp(a) can be more potent in the formation of atherosclerosis throughout the early to progressive stages of atherosclerosis than native Lp(a). These findings warranted further evaluation of the significance of oxLp(a) in the clinical settings.

The carotid arterial intima-media thickness (CIMT) is frequently used in the clinic as one of the most representative surrogate measures of cardiovascular disease (CVD) risk [15,16]. Although we have previously reported a non-significant correlation between the serum oxLp(a) and CIMT, the study population in that report included only healthy young females (mean age: 22 years) all of whom had very low CIMT levels (mean: 0.40 mm) [7]; therefore, it was difficult to assess the correlation. Taking the background into account, the aim of the present study was to investigate the relationship between the levels of serum oxLp(a) and CIMT, in comparison to the serum Lp(a) levels, among asymptomatic middle-aged and older subjects.

## Subjects and Methods

A total of 136 Japanese subjects (mean age; 64 years, range; 40-86 years) were enrolled in this study. The eligibility criteria were: 1) asymptomatic, 2) without pregnancy, 3) neither regularly drank alcohol nor currently smoked, 4) drug-free (including oral contraceptives and over-the-counter drugs such as antioxidant agents), 5) without any history of cardio/cerebrovascular, thyroid, kidney or liver diseases. The Jichi Medical University ethics committee approved the study, and each subject gave informed consent.

The body mass index (BMI), seated systolic blood pressure (SBP)/diastolic blood pressure (DBP) in the upper-arm, plasma glucose and serum lipids/lipoproteins, including Lp(a) and oxLp(a), were measured. Blood was sampled from the antecubital vein after an overnight fast. The samples were immediately evaluated for glucose and lipids, and the serum samples for evaluation of Lp(a) and oxLp(a) were frozen at -80°C until they were used for measurement (within 3 months). Glucose and lipids (LDL cholesterol [LDL-C], high-density lipoprotein cholesterol [HDL-C] and triglycerides [TG]) were measured enzymatically, and the serum Lp(a) was measured by an ELISA system (Shino-test Co. Ltd., Japan) [17]. The assay system for Lp(a) uses only a monoclonal antibody to a non-repeated segment of apo(a), kringle-IV type 5, as both the solid-phase antibody and the detecting capture antibody [17]. In addition, the serum oxLp(a) level was quantified by a sandwich ELISA system using an oxLp(a)-specific monoclonal antibody (161E2) as both the solid-phase antibody and the detecting capture antibody (we think that the use of the same antibody can improve the specificity of the detection, because the specific epitope site is present at ≥ 2 locations in all apo(a) molecules), as previously described [5,7]. This monoclonal antibody has been proven to react with only oxLp(a), but not native Lp(a) and LDL [5]. For the measurement, the serum samples were placed in each well of the Nunc-polystyrene microplates coated with an anti-oxLp(a) monoclonal antibody. The plates were incubated for 1 hour at room temperature, and after washing, were incubated for 1 hour at room temperature with anti-oxLp(a) monoclonal antibody labelled with a peroxidase conjugate. After washing, 3,3',5,5'-tetra-methylbenzidine was added to each well, and the enzymatic reaction was thereafter carried out for 30 minutes at room temperature. After stopping the reaction, the absorbance was read at 450 nm. The concentration of oxLp(a) was calculated based on the concentration of the bovine serum albumin (BSA)-peptide that contributed 16 peptides per 1 molecule of BSA as a standard [5]. The intra- and inter-assay coefficients of variation were 1.2% and 5.0%, respectively.

The CIMT of the common carotid arteries was measured ultrasonographically by a 10-MHz linear type B-mode probe (Aloka Co. Ltd., Japan). The CIMT, bilaterally measured in segments free of plaque (one at the thickest site and another at two other points [1 cm upstream and 1 cm downstream from the thickest site]), was averaged for 3 measurements.

The data are expressed as the means ± standard deviations or medians plus interquartile range. The correlations between the CIMT and the other variables, including Lp(a) and oxLp(a), were examined by Pearson's correlation test as well as a multiple linear regression analysis adjusted for the measured variables (age, gender, BMI, SBP, LDL-C, HDL-C, TG, glucose). Only the SBP was entered into a multiple linear regression analysis model because of its close collinearity with the DBP (r ≥ 0.7). In the multiple linear regression analysis, the values of TG, Lp(a), oxLp(a) and CIMT were calculated after a log-transformation because of their skewed distribution. In addition, the tertile categories of the serum Lp(a) or oxLp(a) levels as an explanatory variable and carotid atherosclerosis (defined as a CIMT of > 1.0 mm [15]) as a criterion variable were entered into a multivariate logistic regression model to estimate the odds ratio (OR) and 95% confidential interval (CI) for carotid atherosclerosis. The crude OR and OR adjusted for the measured variables (age, gender, BMI, SBP, DBP, LDL-C, HDL-C, TG, glucose) were determined. A P value of ≤ 0.05 was considered to be significant.

## Results

The subjects' characteristics are presented in Table 1 and the correlations between Lp(a), oxLp(a) and the other variables are listed in Table 2. A simple correlation test showed a significant and positive correlation between Lp(a) and oxLp(a) (r = 0.509, P < 0.0001). As shown in Table 2, a simple correlation test showed a significant and positive correlation between the patient age and CIMT, as well as between the SBP and CIMT. There was a significant and positive correlation between the oxLp(a) and CIMT, while there was a non-significant correlation between the Lp(a) and CIMT. Subsequently, a multiple linear regression analysis adjusted for the measured variables revealed that there was a significant, independent and positive correlation between the CIMT and oxLp(a), but not Lp(a) level, in addition to a significant, independent and positive correlation between the patient age and CIMT, as well as between the SBP and CIMT. We observed a weaker correlation between the CIMT and the other variables, such as LDL-C, relative to the correlation between the CIMT and oxLp(a), in the multiple linear regression analysis.

**Table 1:** The clinical characteristics of the study subjects

Variable	Levels
Age, years	64 ± 11
Male/female, n	61/75
Body mass index, kg/m^2^	24.7 ± 3.8
Systolic blood pressure, mmHg	129 ± 14
Diastolic blood pressure, mmHg	79 ± 10
Low-density lipoprotein cholesterol, mmol/L	2.98 ± 0.75
High-density lipoprotein cholesterol, mmol/L	1.41 ± 0.38
Triglycerides, mmol/L	1.33 (0.97-1.95)
Glucose, mmol/L	6.44 ± 2.19
Lp(a), μmol/L	120 (70-230)
Oxidized Lp(a), nmol/L	0.06 (0.03-0.12)
CIMT, mm	0.7 (0.6-0.9)

CIMT: carotid artery intima-media thickness. The data are expressed as the means ± standard deviations, medians plus interquartile range (for triglycerides, Lp(a), oxidized Lp(a) and CIMT) or subject number.

**Table 2:** The correlations between the CIMT and Lp(a) or oxidized Lp(a) level

		For Lp(a)	For oxidized Lp(a)
		
Variable	Simple (P)	Multi-adjusted (P)	Multi-adjusted (P)
Age, years	0.415 (< 0.0001)**	0.426 (< 0.0001)**	0.423 (< 0.0001)**
Gender, male	0.072 (0.402)	0.085 (0.297)	0.058 (0.468)
Body mass index, kg/m^2^	0.039 (0.652)	- 0.019 (0.809)	- 0.015 (0.851)
Systolic BP, mmHg	0.253 (0.003)**	0.230 (0.005)**	0.223 (0.006)**
Diastolic BP, mmHg	0.132 (0.124)	-	-
LDL cholesterol, mmol/L	- 0.018 (0.831)	0.106 (0.208)	0.100 (0.224)
HDL cholesterol, mmol/L	- 0.001 (0.993)	- 0.006 (0.943)	- 0.019 (0.822)
Triglycerides, mmol/L	- 0.043 (0.616)	0.015 (0.872)	0.024 (0.792)
Glucose, mmol/L	0.062 (0.476)	0.021 (0.809)	0.031 (0.708)
Lp(a), μmol/L	0.011 (0.901)	0.041 (0.625)	-
Oxidized Lp(a), nmol/L	0.208 (0.015)*	-	0.202 (0.010)**

CIMT: carotid artery intima-media thickness, BP: blood pressure, LDL: low-density lipoprotein, HDL: high-density lipoprotein. Simple: Pearson's correlation coefficient (r), multi-adjusted: a multiple linear regression coefficient (β). The CIMT, triglycerides, Lp(a) and oxidized Lp(a) levels were calculated after a log-transformation. Significance level: * P ≤ 0.05, ** P ≤ 0.01.

In addition, a logistic regression analysis for carotid atherosclerosis using the tertile of the Lp(a) or oxLp(a) levels was carried out. There were 8 subjects with carotid atherosclerosis in the 1^st ^tertile of Lp(a) (< 83 μmol/L [n = 46]), 11 subjects with carotid atherosclerosis in the 2^nd ^tertile of Lp(a) (83-192 μmol/L [n = 45]) and 8 subjects with carotid atherosclerosis in the 3^rd ^tertile of Lp(a) (> 192 μmol/L [n = 45]). The crude OR (95%CI) of Lp(a) was 1.0 (reference) in the 1^st ^tertile, 1.53 (0.55-4.27, P = 0.41) in the 2^nd ^tertile and 1.03 (0.35-3.02, P = 0.96) in the 3^rd ^tertile. The multivariate-adjusted OR (95%CI) of Lp(a) was 1.0 (reference) in the 1^st ^tertile, 1.80 (0.54-6.06, P = 0.34) in the 2^nd ^tertile and 1.08 (0.31-3.82, P = 0.91) in the 3^rd ^tertile. On the other hand, there were 5 subjects with carotid atherosclerosis in the 1^st ^tertile of oxLp(a) (< 0.03 nmol/L [n = 46]), 8 subjects with carotid atherosclerosis in the 2^nd ^tertile of oxLp(a) (0.03-0.08 nmol/L [n = 45]) and 14 subjects with carotid atherosclerosis in the 3^rd ^tertile of oxLp(a) (> 0.08 nmol/L [n = 45]). The crude OR (95%CI) of oxLp(a) was 1.0 (reference) in the 1^st ^tertile, 1.77 (0.53-5.90, P = 0.35) in the 2^nd ^tertile and 3.70 (1.21-11.38, P = 0.02) in the 3^rd ^tertile. The multivariate-adjusted OR (95%CI) of oxLp(a) was 1.0 (reference) in the 1^st ^tertile, 2.72 (0.73-10.18, P = 0.14) in the 2^nd ^tertile and 3.60 (1.01-12.92, P = 0.048) in the 3^rd ^tertile. Therefore, the subjects with the highest tertile of oxLp(a), but not Lp(a), had a significantly and independently high OR for the presence of carotid atherosclerosis.

## Discussion

The present study showed that the serum oxLp(a) level was a significant, independent and positive measure of carotid atherosclerosis, as assessed by the CIMT, in comparison to the serum Lp(a) level, in asymptomatic subjects. A weak correlation between the Lp(a) and CIMT, and a relatively low level of Lp(a) (compared to an earlier study of a general Japanese population [18]) were observed in this study. This may be, in part, due to the studied population, all of whom were asymptomatic and of possibly good health, as well as due to the Lp(a) detection method using the antibody to a non-repeated segment of apo(a), which is not affected by apo(a) size polymorphisms [17]. While the impact of oxLp(a) on carotid atherosclerosis was clear in the subjects with the highest tertile of oxLp(a), the circulating level of oxLp(a) was lower than that of Lp(a) and the correlation between the oxLp(a) and CIMT was not very strong as an overall finding. Therefore, the clinical relevance of the results will need to be confirmed in further studies. However, this finding obtained by the use of the CIMT is valuable because carotid atherosclerosis is a well-known clinical predictor of the CVD risk [15,16]. Of note, the present findings suggest that high serum levels of oxLp(a) may be associated with accelerated atherosclerosis, which provides important information for obtaining a better understanding of the different atherogenic roles played by oxLp(a) in comparison to Lp(a), in addition to the earlier knowledge about the clinical associations among Lp(a), oxLp(a) and atherosclerotic manifestations [6,8].

The precise reason for the association between the serum oxLp(a) and CIMT in this study was unclear, although a possible mechanism has been suggested. One experimental study reported the generation of O^2- ^(promoting oxidative stress) in oxLp(a)-treated rabbit renal arteries, which could lead to endothelial dysfunction (this occured in the oxLp(a)-, but not native Lp(a)-, treated rabbits) [11]. One clinical study showing a significant and positive correlation between the oxLp(a) and pulse wave velocity in hypertensive patients assumed that the oxidative status of hypertension could contribute to that correlation [6]. Another histochemical study on human carotid arteries reported the presence of oxLp(a) in the endothelial cells and subendothelial layers, indicating that native Lp(a) is initially oxidized or that oxLp(a) is initially taken up from the blood into the endothelial cells and subendothelial layers [8]. Moreover, that study implicated oxLp(a) as having more involvement, relative to native Lp(a), in the progression of arthrosclerosis, based on the abundant presence of oxLp(a) in the synthetic phase vascular smooth muscle cells [8]. In general, oxidative stress induces increases in the CIMT, and the increased CIMT can further enhance the oxidative stress, leading to a vicious cycle (this cycle may be essentially pronounced during the progressive stage of arthrosclerosis) [19]. In addition, it is thought that Lp(a) can be atherogenic in relation to oxidized molecules, such as oxidized phospholipids, under the oxidative milieu, where the oxidation of Lp(a) may be promoted [4]. In this context, we hypothesize that once oxLp(a) is present, it induces an oxidative condition, and this condition increases the CIMT, and a vicious cycle of oxLp(a)-CIMT elevation occurs as a result.

There are several limitations to this study. The study population was restricted to asymptomatic subjects, not an obviously diseased population. The cross-sectional design of the study did not clearly elucidate the cause-and-effect on the results. In addition, the residual confounding factors that can affect the oxidative status and/or the oxidation of lipoproteins (i.e., dietary factors) were not available in the study. Therefore, future studies with different populations, prospective designs and the inclusion of many confounders will be necessary to establish our findings.

## Conclusions

In summary, the serum oxLp(a) was more significantly, independently and positively correlated with the CIMT than the serum Lp(a), in asymptomatic middle-aged and older subjects. This finding seems to be important for obtaining a better understanding of the different atherogenic roles played by oxLp(a) in comparison to Lp(a) in the pathogenesis of atherosclerosis. Further studies are required to determine the observed relationship and to clarify the biological mechanisms involved in this relationship.

## Competing interests

The authors declare that they have no competing interests.

## Authors' contributions

All authors contributed to the intellectual development of this work, and approved the final manuscript. KK, SY, TY and NT analyzed the data. KK and SY searched the literature and wrote the draft paper. NT and IS provided critical corrections to the manuscript.
